# Generation and differentiation of induced pluripotent stem cells reveal ankylosing spondylitis risk gene expression in bone progenitors

**DOI:** 10.1007/s10067-016-3469-5

**Published:** 2016-11-18

**Authors:** Gerlinde Layh-Schmitt, Shajia Lu, Fatemeh Navid, Stephen R. Brooks, Emily Lazowick, Kathryn M. Davis, Cristina Montagna, Massimo Gadina, Robert A. Colbert

**Affiliations:** 1 Pediatric Translational Research Branch, NIAMS, NIH, Bldg. 10/CRC, Rm 1-5132, 10 Center Drive MSC 1102, Bethesda, MD 20892 USA; 2 Translational Immunology Section, Office of Science and Technology, NIAMS, NIH, Bethesda, MD USA; 3 Biodata Mining and Discovery Section, Office of Science and Technology, NIAMS, NIH, Bethesda, MD USA; 4 Department of Genetics, Albert Einstein College of Medicine, Bronx, NY USA

**Keywords:** Bone, Rheumatic diseases, Spondyloarthritis, Tissues or models

## Abstract

Axial spondyloarthritis (axSpA), which encompasses ankylosing spondylitis, is a complex genetic disease. Aberrant bone formation is a key feature of pathogenesis that can lead to ankylosis of the spine. Our objective is to determine, whether genes whose variants confer susceptibility to AS are expressed in bone progenitors like mesenchymal stem cells (MSCs). Since MSCs from bone marrow is difficult to obtain, we first examined, whether MSCs can be derived from induced pluripotent stem cells (iPSCs). Dermal fibroblasts of two axSpA patients and one healthy control were reprogrammed into iPSCs using a Sendai virus vector encoding pluripotency genes. Pluripotency of iPSCs was examined by embryoid body formation and by testing for stem cell specific gene and protein expression using RT-PCR and immuno fluorescence. iPSCs were differentiated into MSCs by a TGFß inhibitor. MSCs were characterized by flow cytometry using lineage specific antibodies and by their capacity to develop into chondrocytes, adipocytes, and osteoblasts in lineage-specific medium. RNA-seq was applied to determine genome-wide gene expression patterns in MSCs, iPSCs, and blood. We show for the first time, that expression levels of several AS susceptibility genes (EDIL3, ANO6, HAPLN1, ANTXR2) involved in bone formation are significantly elevated in MSCs (2–15-fold; *p* ≤ 0.05) compared to blood or iPSCs and demonstrate that iPSC-derived MSCs can be differentiated into osteoblasts, chondrocytes, and adipocytes. We conclude, MSCs generated from patient fibroblast-derived iPSC lines are useful tools for studying functional genomics of risk genes associated with bone formation in AS pathogenesis.

**Electronic supplementary material:**

The online version of this article (doi:10.1007/s10067-016-3469-5) contains supplementary material, which is available to authorized users.

## Introduction

Ankylosing spondylitis (AS) is characterized by inflammation of the sacroiliac joints, vertebral bodies and facet joints, entheses, and peripheral joints, as well as extra-articular sites such as the gastrointestinal tract [1]. Axial arthritis is associated with bone marrow inflammation, vertebral trabecular bone loss, and progressive bone formation between vertebral bodies (syndesmophytes), and in facet joints that can result in ankylosis leading to severe disability. Axial spondyloarthritis (axSpA) encompasses AS and earlier stages of disease when radiographic damage to the sacroiliac joints is limited or absent, but bone marrow edema is often prominent [2]. Genome-wide association studies have shown, that genetic susceptibility to AS is complex with 40 genes or genetic regions implicated [3]. However, mechanisms underlying the pathogenesis of axSpA/AS, and in particular the involvement of specific cell types and events that lead to bone marrow edema, loss of trabecular bone, and aberrant bone formation in the spine, are still poorly understood [4, 5]. Evidence that cytokines such as TNFα [6], IL-23 [7], and IL-17 [8] are expressed in axial skeletal tissue from AS patients has provided a rationale for current therapies [9–11]; but the mechanisms driving their production and their effects on bone formation require further study. Progress in this area has been hampered by limited access to cell types that contribute directly to disease pathogenesis, such as hematopoietic progenitors, mesenchymal cells, and their derivative osteoblasts and adipocytes.

In this study, our goal was to explore the feasibility of reprogramming skin fibroblasts into pluripotent stem cells (induced pluripotent stem cells, or iPSCs) that can be differentiated into disease-relevant cell types allowing us to examine axSpA/AS pathogenesis. We demonstrate this by differentiating iPSCs into mesenchymal stem cells (MSCs), and then further differentiating MSCs into cells capable of forming bone, cartilage, and fat. Importantly, using genome-wide gene expression analysis, we show for the first time that the expression of certain AS risk genes is enriched in bone progenitors. These studies demonstrate that iPSCs may be a useful tool to better understand the pathogenesis of AS.

## Methods

### Generation of induced pluripotent stem cells

Fibroblasts were grown from ‘shave’ skin biopsies obtained from three subjects enrolled in an Institutional Review Board approved natural history protocol at the NIH Clinical Center. One subject was a healthy donor (HC1), and two subjects (P1 and P2) met criteria for axSpA [2]. Fibroblasts were cultured in DMEM supplemented with 10% FBS (Gibco/Life Technologies) at 37 °C and 5% CO_2_, and reprogrammed using a Sendai virus vector encoding OCT4, SOX2, KLF4, and MYC (CytoTune-iPS reprogramming kit, Invitrogen) [12, 13]. Duplicate iPSC lines were generated independently in two laboratories (L1 and L2) for one axSpA patient (P1L1 and P1L2) and one healthy control (HC1L1 and HC1L2). One additional iPSC line was generated from P2 (P2L2). Detailed methods for iPSC generation are provided in Supplementary Methods.

### Pluripotency and stability analyses

Expression of Sendai virus transgenes and endogenous pluripotency genes was evaluated by real-time (RT) PCR (RT-PCR). Embryoid body (EB) formation was examined to confirm pluripotency of iPSC lines, and chromosomal stability was determined by karyotyping. Detailed descriptions are provided in Supplementary Methods.

### Differentiation of iPSCs into MSCs

iPSC lines were maintained and propagated in E8 medium (Gibco/Life Technologies) [14]. Between passage 20 and 25, all five lines were transferred into 10 cm^2^ culture dishes to differentiate them into MSCs. After two more days of culture (maximum confluence 50%), medium was replaced with TGFβ-free E6 medium (Gibco/Life Technologies) containing 10 μM SB-431542 (Tocris), a TGFβ inhibitor. Cells were cultured for another 6 days with one change of medium at day 3. Thereafter, cells were maintained in MEM-α medium with 15% FBS (Hyclone) (non-osteogenic, or OS−) to proliferate MSCs. Cells were split using TrypLE (Gibco/Life Technologies).

### Differentiation of iPSCs into monocytes/macrophages

iPSCs were differentiated into monocytes/macrophages in four stages using defined cytokine cocktails as described [15] in detail in Supplementary Methods. Stage 4 cells (monocytes) were differentiated into osteoclasts by culturing them in DMEM (Gibco/Life Technologies) with 10% FBS, 20 ng/ml MCSF, and 100 ng/ml RANKL (both from Peprotech). After 15 days, cells were fixed with 10% formaldehyde for 5 min and stained for osteoclast-specific tartrate-resistant acid phosphatase (TRAP) activity using a TRAP staining kit (Kamiya). TRAP-positive cells with ≥3 nuclei were considered osteoclasts.

### Immunostaining and microscopy of iPSCs and EBs

To determine expression of cell type-specific proteins, cells were fixed in PBS containing 4% paraformaldehyde for 20 min followed by treatment with 10% goat serum in 0.1% Triton X-100 for 10 min (Sigma-Aldrich). Cells were rinsed with PBS and then incubated with iPSC-specific antibodies: anti-OCT4 (Alexa-488 labeled, Millipore), anti-TRA-1-60 (FITC labeled; Millipore clone TRA-1-60), and anti-SSEA-4 (FITC labeled; Biolegend); all antibodies were diluted 1:50 in PBS. Paraformaldehyde-fixed EBs derived from iPSCs were examined for expression of germ layers using an Alexa Fluor 488-labeled monoclonal antibody against βIII tubulin (βIII TUB) (Covance) at 1:200 dilution, anti-smooth muscle actin (α-SMA) (Millipore) at 1:100 dilution and a polyclonal rabbit anti-human alpha-1-fetoprotein (AFP) antibody (Dakocytomation) at 1:500 dilution in PBS. Cy3 goat anti-rabbit IgG (Invitrogen) (1:500 dilution) and Alexa Flour 488 goat anti-mouse IgG (H+L) (Invitrogen) (at 1:100 dilution) were used as secondary antibodies. Nuclei were stained with DAPI (Invitrogen). Images were taken with a Nikon Eclipse TE 300 microscope using 10× or 20× objectives.

### Flow cytometry of iPSC-derived MSCs and monocytes

To evaluate cell surface expression of specific markers, iPSC-derived MSCs were dissociated with TrypLE Select, washed twice with FACS buffer (PBS, 0.5% BSA), and blocked with FcR blocking reagent (BD) for 10 min at 4 °C before incubation with antibodies using the BD Stemflow human MSC analysis kit. The panels for multicolor analysis of human MSCs included the conjugated antibody cocktail: FITC-CD90, PerCP-Cy™5.5-CD105, APC-CD73, PE-CD44 for positive staining, and the MSC-negative antibody cocktail: PE-CD45, PE-CD34, PE-CD11B, PE-CD19, and PE-HLA-DR. To assess monocyte surface markers, cells in Stage 4 were treated with FcR blocking reagent and then incubated with APC-CY7-CD14, APC-HLA-DR, FITC-CD80, FITC-CD86, APC-CX3CR1 (eBiolegend). To examine function, Stage 4 cells were incubated with FITC labeled beads (Life Technologies) for 30 min and phagocytosis was measured by FACS analysis. Data were collected using BD FACSCanto RUO and analyzed using FlowJo software (Treestar).

### Gene expression analysis

Gene expression in iPSCs, MSCs and peripheral blood cells was determined by RNA-seq. RNA was isolated from iPSCs and MSCs using the Direct-zol RNA MiniPrep kit (Zymo Research, Irvine, CA, USA). Peripheral blood was collected in PAXgene Blood RNA tubes, and RNA was isolated using the PAXgene RNA kit (Qiagen). RNA quality was analyzed using the Agilent RNA 6000 Nanokit (Agilent Technologies), and samples with an RNA integrity number greater than 8 were used. Complementary DNA libraries for RNA-seq were prepared based on standard procedures (TruSeq RNA sample preparation guide) published by Illumina. Adapter ligation using the Mondrian SP cartridge (Nugen) was performed on the Mondrian SP workstation (Nugen); library amplification and purification were carried out according to the manufacturer’s instructions. Sequencing was performed using an Illumina HiSeq2500 ultra-high-throughput sequencing system. Reads per kilobase million (RPKM) values represent the number of mapped reads for a gene determined from the RNA-seq run, normalized to the length of the gene and the total number of reads from that run. Five iPSC lines and five MSC lines (including replicate derived HC1 and P1) were analyzed along with six different whole blood (PAXgene) samples from healthy controls using principal components analysis (PCA). Expressed genes were defined as those with RPKMs ≥1 in at least one sample. Differentially expressed genes across all samples (iPSCs, MSCs, and blood) shown in heat maps were defined as those with a coefficient of variation (standard deviation divided by the mean) that was greater than 0.7. Relative expression was determined by subtracting the mean RPKM from the RPKM value for the gene of interest, and dividing by the standard deviation: [relative expression = (value_RPKM_ – mean_RPKM_)/std. deviation]. RPKM, PCA, and heat maps were generated using Partek GS 6.6. To analyze relevant pathways represented in the three cell types, ingenuity pathway analysis was applied to differentially expressed genes defined by ANOVA with a fold change of five or more, and a *q* value less than or equal to 0.1. In osteoblast differentiation experiments, gene expression was measured using nanostring technology according to the manufacturer’s instructions.

### Differentiation of iPSC-derived MSCs into chondrocytes, adipocytes, and osteoblasts

Detailed methods for differentiation of MSCs into chondrocytes, adipocytes, and osteoblasts are described in Supplementary Methods.

### Co-culture of MSCs and monocytes

To examine whether iPSC-derived MSCs were capable of promoting osteoclast development, we performed co-culture experiments in high-bind 6-well tissue culture plates (Costar). Three millimeters of 1 × 10^6^ monocytes/ml (isolated from a healthy donor by apheresis) in MEM-α (Gibco/Life Technologies) with 15% FBS (Hyclone) were seeded onto a monolayer of iPSC-derived MSCs (10^5^ cells/well). As controls, MSCs and monocytes alone were cultured separately. Cell cultures were fed every 2–3 days, and after 15 days, were assessed for TRAP-positive osteoclasts as described above.

## Results

### Generation of iPSCs

**Fig. 1.**
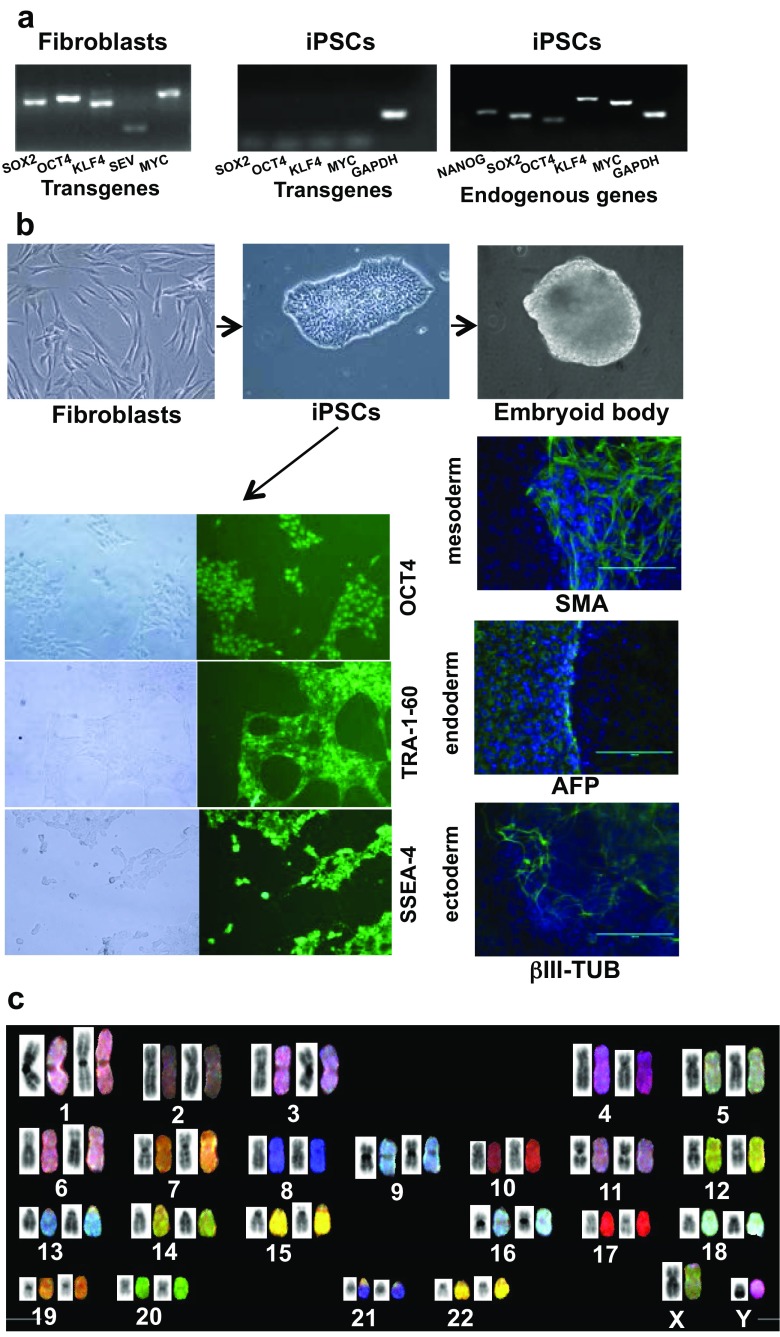
Generation and characterization of iPSCs. **a** RT-PCR analysis of Sendai virus-encoded transgenes (SOX2, OCT4, KLF4, MYC) in dermal fibroblasts 7 days after transduction (*left panel*) (SEV, Sendai virus), and then after 21 days (11 passages) (*middle panel*). RT-PCR analysis of endogenous pluripotency genes in iPSCs after 21 days (*right panel*). (Primer specificity enabling discrimination between virus-encoded vs. endogenous genes is described in Methods.) **b** Images of fibroblasts, iPSCs, and an embryoid body derived from iPSCs. Immunofluorescence microscopy showing expression of pluripotency proteins OCT4, TRA-1-60, and SSEA-4 in iPSCs (*left*), and proteins specific for each germ layer present in embryoid bodies (*right*). Representative data from P1L1 are shown. SMA, α-smooth muscle actin; AFP, α-fetoprotein; βIII-TUB, βIII tubulin. **c** Spectral karyotyping of iPSCs. Images for a minimum of ten metaphase spreads were acquired with an Olympus BX61 microscope (Olympus, Shinjuku Tokyo, Japan) equipped with a spectracube and analyzed using the HiSKY software. The image shown was derived from P1L1 iPSC chromosomes at passage 31. Similar results were obtained at passage 40 (not shown)

Dermal fibroblasts transduced with Sendai virus encoding reprogramming genes were cultured on feeder cells in iPSC medium for 12–21 days. Viral transgenes were expressed at day 7 after transduction (Fig. 1a, left panel), but not 21 days later (passage 11) (Fig. 1a, middle panel), when endogenous pluripotency gene expression was apparent (Fig. 1a, right panel). Expression of stem cell specific proteins SSEA-4, TRA-1-60, and OCT4 was documented by immunofluorescence microscopy (Fig. 1b, left panel). EBs generated from iPSCs exhibited all three germ layers based on expression of the endodermal marker AFP, the mesodermal protein SMA, and the ectodermal marker βIII-TUB (Fig. 1b, right panel) [12, 16]. Normal karyotype and chromosomal stability of iPSCs were verified by spectral karyotyping at passages 31 and 40 (Fig. 1c).

### Generation of MSCs

**Fig. 2.**
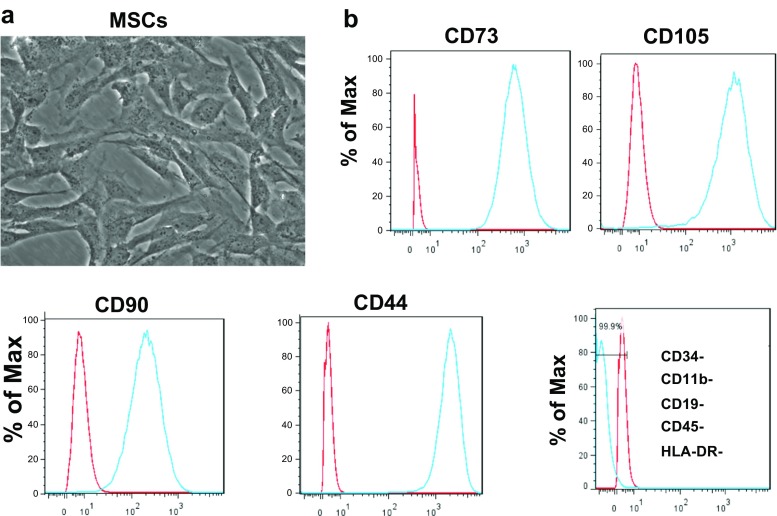
Differentiation of iPSCs into MSCs. iPSCs were cultured for 6 days in TGFβ-free medium in the presence of TGFβ inhibitor. **a** Morphology of iPSC-derived MSCs under standard light microscopy (original magnification 20×). **b** Flow cytometric analysis of MSCs using antibodies against known surface markers CD73, CD105, CD90, and CD44. Staining for other lineage markers (*bottom right*). *Red line*, trace for isotype control; *blue lin*e, cells stained with antibodies as indicated. Representative results from P1L1 are shown in **a** and **b**

To generate MSCs, iPSC lines were cultured in TGFβ-free medium supplemented with a TGFβ inhibitor (SB-431542) for 6 days. In addition to characteristic morphology (Fig. 1a), MSCs exhibited expression of typical markers (CD73, CD105, CD44, and CD90), but were negative for other lineage markers (CD34, CD11b, CD19, CD45, HLA-DR) (Fig. 2b). Differentiation into MSCs was highly efficient with 96–99% of cells staining positive for specific markers (Fig. 2b).

### Gene expression analysis of MSCs and iPSCs

**Fig. 3.**
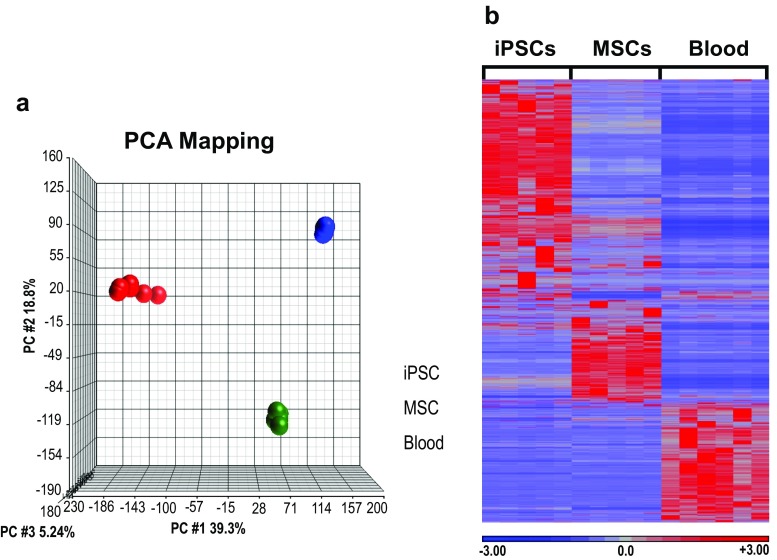
Differentially expressed genes in iPSCs, MSCs and blood cells. RNA isolated from iPSC and MSC lines, and peripheral blood from healthy controls was analyzed by RNA-seq to assess cell type-specific gene expression patterns. **a** Gene expression in MSCs was compared with iPSCs and peripheral blood samples using RNA-seq and principle components analysis (PCA) of expression differences. Three-dimensional plot shows three clusters with each cluster containing all five samples generated from iPSC lines (*blue*), MSC lines (*green*), and peripheral blood cells (red). (Tight clustering of iPSCs (*blue*) and MSCs (*green*) prevents visualization of individual samples.) **b** Heat map generated based on RPKM values for all differentially expressed genes in iPSCs, MSCs, and blood as defined in Methods. *Red* represents increased and *blue* decreased expression, and the color intensity reflects the −log_10_ of the *P* value for each gene

Genome-wide gene expression in iPSCs, iPSC-derived MSCs, and peripheral blood cells was assessed by RNA-seq. PCA revealed major differences in gene expression between iPSCs, MSCs, and whole blood, but tight clustering of the same cell types derived from different individuals (Fig. 3a). To identify differentially expressed genes RPKM values were compared. Statistically significant differences are displayed as a heat map (Fig. 3b), where red represents increased and blue decreased expression (relative to the mean across all samples), and the color intensity reflects the number of standard deviations above or below the mean. Highly expressed genes were largely distinct for each cell type, with only a few regions of overlap. All iPSC lines expressed stem cell specific genes (NANOG, OCT-4, SOX2, DPPA2, TERT, MYC, ACTC1, CCNE1, CD9, CDH2, FOXD3, GABRB3, GJA1, NODAL) [16–19], consistent with their pluripotency as shown in Fig. 1. The major pathways represented by the differentially expressed genes were determined using ingenuity pathway analysis (Supplementary Table 1). This revealed the ‘role of Oct4 in pluripotency’ and ‘transcriptional regulation of embryonic stem cells’ in iPSCs. In MSCs, pathways involved in ‘actin and collagen’ expression and ‘endocytosis signaling’ were prominent. Peripheral blood reflected specific immune cell types and antigen presentation.

### Ankylosing spondylitis risk gene expression

**Fig. 4.**
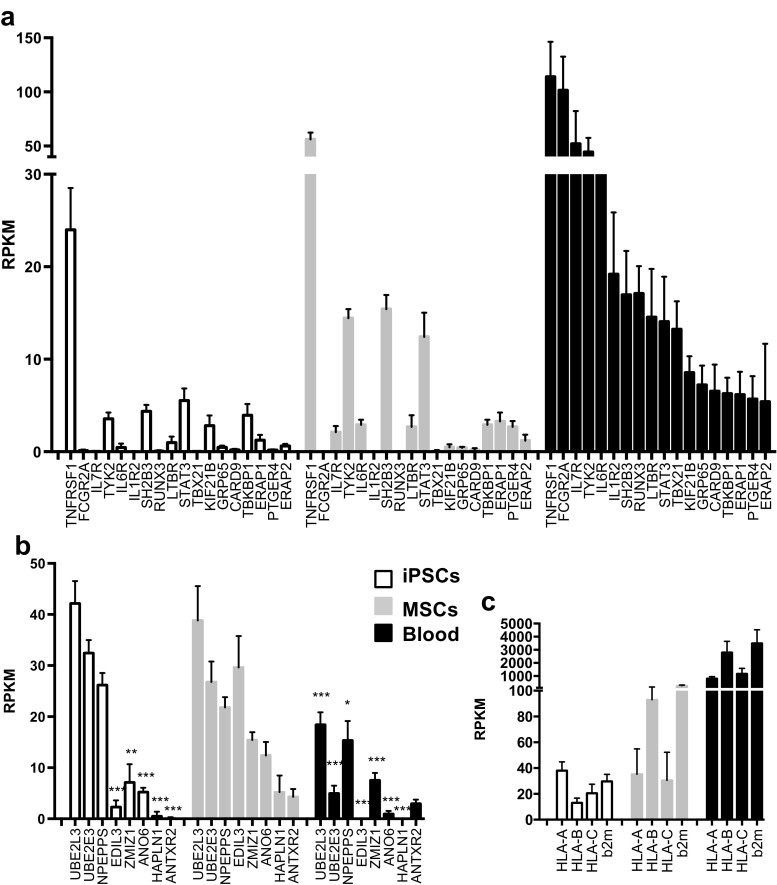
AS risk gene expression in iPSCs, MSCs, and peripheral blood. AS risk genes identified from genome-wide association studies were compared for relative expression in 5 iPSC and 5 iPSC-derived MSC lines, and blood from six healthy donors using RNA-seq data. Only genes with an RPKM value >2 in at least one cell type are shown. **a** AS risk genes enriched in peripheral blood. **b** AS risk genes enriched in MSCs. *Asterisks* indicate genes, which are significantly lower expressed in iPSCs and blood compared to MSCs. **c** HLA-A, B, C, and β_2_m expression. Data represent average RPKM values with the *error bars* indicating standard error of the mean. *Open bars* indicate iPSCs, *shaded bars* are MSCs, and *solid bars* represent peripheral blood

To determine whether known AS risk genes [3] were expressed in reprogrammed cells, we examined iPSCs and MSCs derived from the iPSCs, using peripheral blood for comparison. By using a cutoff of RPKM ≥2, we found 27 AS risk genes (RUNX3, KIF21B, PTGER4, ERAP1, ERAP2, CARD9, LTBR, STAT3, TNFRSF1A, NPEPPS, TBkBP1, TBX21, IL6R, FCGR2A, UBE2E3, UBE2E3, NKX2-3, ZMIZI, SH2B3, GPR65, TYK2, IL7R, ANTXR2, HAPLN1, EDIL3, ANO6, HLA-B) expressed in one of the examined cell types. In peripheral blood, 18 AS risk genes (RUNX3, KIF21B, PTGER4, ERAP1, ERAP2, CARD9, LTBR, STAT3, TNFRSF1A, TBkBP1, TBX21, IL6R, FCGR2A, GPR35, NKX2-3, SH2B3, GPR65, TYK2, IL7R, HLA-B) were expressed to a much greater extent (>2-fold higher) than in MSCs or iPSCs (Fig. 4a, c). TNFRSF1 (encoding the p55 subunit of the type I TNF receptor), SH2B3 (SH2B adaptor protein 3), and STAT3 (signal transducer and activator of transcription 3) were expressed in MSCs at levels comparable (<2-fold difference) to peripheral blood (Fig. 4a). TYK2 (tyrosine kinase-2) was also expressed in MSCs, but about 3-fold less than in whole blood. Interestingly, five AS risk genes (EDIL3, ZMIZ1, ANO6, HAPLN1, and ANTXR2) were expressed at significantly higher levels (2–15-fold) in MSCs relative to either iPSCs or peripheral blood (Fig. 4b). Three additional genes, NPEPPS (puromycin-sensitive aminopeptidase), UBE2L3, and UBE2E3 (both are ubiquitin-conjugating enzymes) were expressed to a greater extent (2–3-fold) in MSCs and iPSCs than in whole blood. HLA class I A, B, and C and β_2_m expression was readily detectable iPSCs and MSCs, but much higher in blood (up to 20–30-fold) (Fig. 4c).

### MSC differentiation into chondrocytes, adipocytes, and osteoblasts

**Fig. 5.**
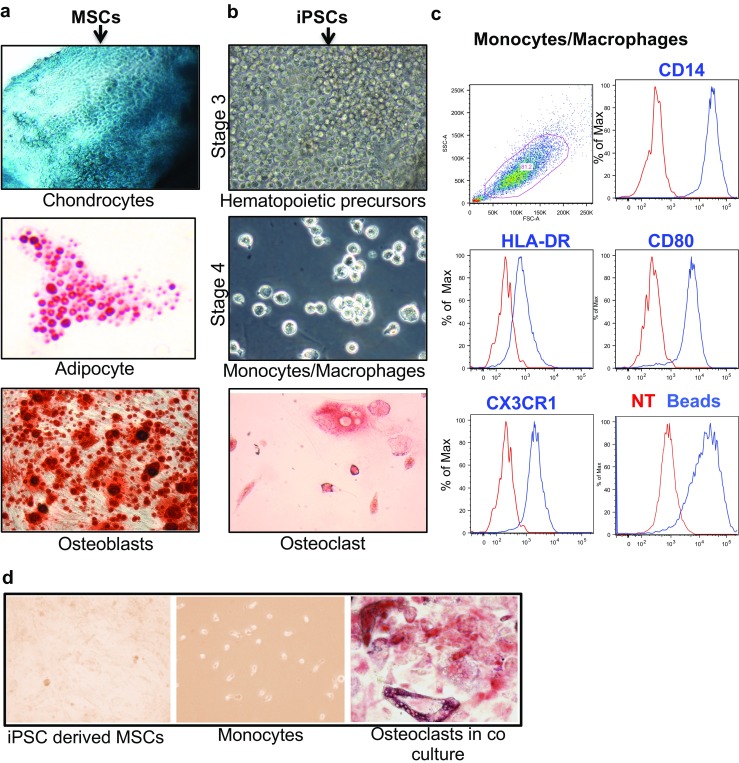
Differentiation of iPSCs into stromal and hematopoietic cells. **a** MSCs derived from iPSCs were differentiated into chondrocytes exhibiting alcian blue positive staining due to aggrecan production (*top panel*); into adipocytes characterized by prominent intracellular fat droplets stained with Oil Red O (*middle panel*); and into osteoblasts as shown by alizarin red staining of mineralized matrix (*bottom panel*). **b** Monocytes/macrophages were generated from iPSCs using cytokine cocktails (*middle panel*) and further differentiated into multi-nucleated osteoclasts by culturing with RANKL in the continued presence of MCSF (*bottom panel*). Osteoclasts were stained for TRAP expression. **c** Monocytes derived from iPSCs (P1L1) were examined for expression of CD14, HLA-DR, CD80, and CX3CR1 by flow cytometry, and their ability to phagocytose fluorescent beads. **d** iPSC-derived MSCs and freshly isolated human blood monocytes were cultured alone or together for 15 days, and then stained for TRAP activity. TRAP-positive multi-nucleated osteoclasts were observed only in co-cultures (*right panel*)

To demonstrate the potential of MSCs to become chondrocytes, adipocytes, or osteoblasts, they were differentiated using cell type-specific conditions. Chondrocytes were apparent after about 3 weeks. The differentiation procedure was highly efficient with the majority of cells staining positive for chondrocyte-specific aggrecan with alcian blue (Fig. 5a, top). Adipocytes were visualized after 21 days using Oil Red O to stain intracellular lipid droplets (Fig. 5a, middle). The efficiency of adipocyte differentiation was about 40% (negative cells not shown). Osteoblasts capable of mineralizing were observed after 21–28 days in osteogenic medium, as shown by alizarin red staining (Fig. 5a, bottom).

### Induced pluripotent stem cell differentiation into monocytes/macrophages

Differentiation of iPSCs into myelomonocytic cells involved several stages, during which different cytokine cocktails were used [15]. The first two stages required about 14 days. At Stage 3, morphologically distinct (round) cells began to develop that likely represented hematopoietic precursors (Fig. 5b, top panel). These cells were expanded by a cocktail of cytokines that included stem cell factor (SCF), FMS-like tyrosine kinase 3 ligand (Flt3L), and IL-3. After 4 days, cells were cultured for an additional 14 days with MCSF resulting in monocytic cells (Stage 4) (Fig. 5b, middle panel). At this stage the cells were virtually 100% monocytes, as shown by staining for CD14, HLA-DR, CD80, and CX3CR1, and their ability to phagocytose beads (Fig. 5c). In addition, these monocytes could be differentiated into osteoclasts using RANKL as shown by the appearance of tartrate-resistant acid phosphatase (TRAP) positive multi-nucleated cells (Fig. 5b, bottom).

### MSC induction of osteoclast formation

To determine whether iPSC-derived MSCs would support osteoclast formation, we co-cultured MSCs with fresh human monocytes derived from the peripheral blood of a healthy donor. After 18 days, multi-nucleated TRAP-positive osteoclasts were readily apparent (Fig. 5d), but absent when monocytes and MSCs were cultured alone.

### Osteoblast mineralization potential

**Fig. 6.**
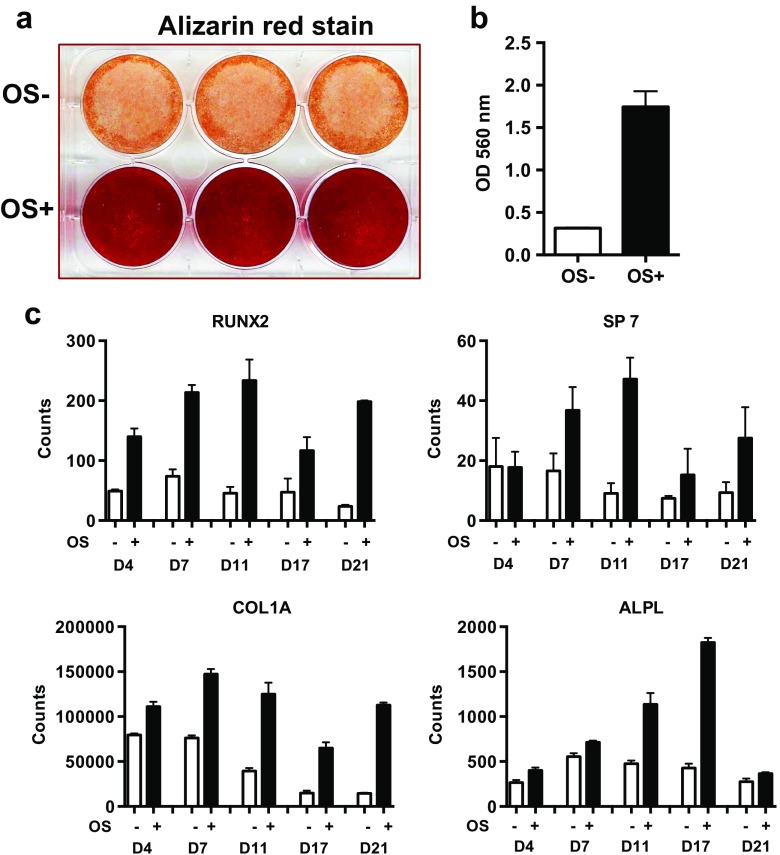
Osteoblast mineralization potential. **a** iPSC-derived MSCs were cultured in non-osteogenic (OS−) or osteogenic (OS+) media for 21 days, and then stained with alizarin red to visualize mineralized matrix. A representative image of alizarin red-stained matrix from patient (P1L1)-derived mineralizing osteoblasts is shown (*triplicate*). **b** Quantification of extracted alizarin red stain from mineralizing osteoblast cultures (P1L1). Quantitative data show triplicate cultures from one experiment that is representative of several. **c** Relative mRNA expression levels of osteogenic genes (normalized to house keeping genes RPL19, HPRT1, PPIA) during osteoblast differentiation of MSCs derived from a patient (P1L1) in OS+ medium in comparison to MSCs cultured in OS− at days 4–21. A representative experiment in triplicates is shown. *Open bars* OS− media, *solid bars* OS+ medium

The potential of iPSC-derived MSCs to differentiate into mineralizing osteoblasts was examined by culturing MSCs in osteogenic OS+ medium (MEM-alpha Gibco/Life Technologies, 15% Hyclone FBS, 10 mM β-glycerophosphate, 50 μg/mL ascorbic acid, 10 nM dexamethasone). In comparison, MSCs were maintained in OS− medium lacking osteogenic additives (MEM-alpha Gibco/Life Technologies, 15% Hyclone FBS). After 21 days, the degree of mineralization was visualized by alizarin red staining (Fig. 6a), as described in detail in Supplementary Methods. Quantitation of extracted alizarin stain revealed a 6–7-fold increase (Fig. 6b) for cells cultured in osteogenic medium. Elevated expression levels of osteogenic genes (RUNX2, SP7 [Osterix], COL1A1 [collagen1A1], and ALPL [alkaline phosphatase]) during osteoblast differentiation were confirmed (Fig. 6c) by nanostring analysis.

## Discussion

Progress in understanding the pathogenesis of structural damage in axSpA/AS has been hampered by several factors, including the anatomic location of affected tissue, the absence of animal models that faithfully reproduce the bone phenotype, and the lack of primary patient-derived cell lineages that mediate osteoproliferation and bone marrow edema. Our initial goal was to reprogram skin fibroblasts by transiently expressing the necessary transcription factors (OCT4, SOX2, KLF4, and MYC) from an exogenous source that convert adult cells into pluripotent stem cells. Pluripotency of iPSCs derived from reprogrammed fibroblasts was demonstrated by standard criteria including the formation of embryoid bodies exhibiting all three germ layers, and differentiation of iPSCs into multiple cell lineages, as well as global gene expression patterns. We generated MSCs from iPSCs, and showed that the MSCs could be further differentiated into osteoblasts, adipocytes, and chondrocytes. IPSCs could also be successfully differentiated into monocytes capable of phagocytosing and forming osteoclasts. We also showed that MSCs were competent to drive osteoclast formation from peripheral blood monocytes when cultured together. It is important to note that two different iPSC lines, and their derivative MSCs, that were generated independently from the same individual fibroblast line did not show significant phenotypic differences based on gene expression patterns or mineralization potential when differentiated into osteoblasts (data not shown). This indicates that technical variability in creating these iPSC lines does not generate major phenotypic differences, which will be important in future studies of inter-individual functional differences.

Mechanisms leading to aberrant bone formation and structural damage in axSpA/AS are poorly understand, but are most likely a consequence of multiple factors including the location of inflammatory lesions, entheseal stress, and the nature of the inflammatory response [1]. Many genes or genetic regions associated with susceptibility to AS broadly implicate antigen presentation, lymphocyte development, immune activation and signaling, ubiquitin-mediated protein regulation, aminopeptidase activity, and other functons in disease [3, 20, 21]. Whether or not AS risk genes directly affect osteoproliferation remains a question of interest. We show here that MSCs express several risk genes (EDIL3, HAPLN1, ANO6, ANTXR2, ZMIZ1) to a greater extent than either peripheral blood or iPSCs, and three others (UBE2L3, UBE2E3, and NPEPPS) are expressed prominently in both iPSCs and MSCs. UBE2L3 and UBE2E3 are ubiquitin-conjugating enzymes with different biological functions. UBE2L3 is involved in NF-κB activation downstream of both TNF and CD40 signaling [22]. In contrast, UBE2E3 promotes nuclear localization of Nrf2 (nuclear factor erythroid 2-related factor 2) [23], which is an antioxidant transcription factor that mediates cellular responses to reactive oxygen species (ROS) [24]. NPEPPS (also known as puromycin-sensitivie aminopeptidase or PSA) protects cells from aggregation-prone misfolded proteins [25], in part by enhancing autophagic clearance [26]. Peptides trimmed by NPEPPS can also access major histocomatibility class I proteins in the ER [27, 28]. Both of these functions could influence the impact HLA-B27 may have on cells [29]. The risk genes whose expression is enriched in MSCs are candidates for involvement in the bone phenotype. EDIL3 and HAPLN1 are adjacent to the AS-associated single nucleotide polymorphism (SNP) (rs4552569) [30]. EDIL3 encodes EGF repeats and discoidin I-like domains protein (also known as DEL1 in mice), which is involved in bone mineralization [31], epithelial-mesenchymal transitioning [32], and IL-17-mediated inflammatory bone loss [33]. HAPLN1 encodes hyaluronan and proteoglycan link protein 1 (also known as CRTL1 for cartilage link protein 1), which is a component of extracellular matrix required for normal cartilage development. SNPs in HAPLN1 have also been associated with spinal osteophyte formation in osteoarthritis [34]. ANO6 encodes anoctamin-6 (also known as TMEM16F), which is a membrane phospholipid scramblase that affects bone mineralization by activating the NCX1 calcium transporter [35]. Moreover, Ano6-deficient mice have skeletal deformities and mineralization defects [36]. ANTXR2 encodes the anthrax toxin receptor 2, which binds LRP6, a Wnt co-receptor involved in bone formation [37]. Finally, ZMIZ1 (zinc finger MIZ-containing 1) is in the PIAS (protein inhibitor of activated STAT) family of proteins which enhances SMAD signaling in response to TGFβ [38]. In addition to possible effects on osteogenesis, a region containing ZMIZ1 has been associated with inflammatory/autoimmune diseases including Crohn’s disease and ulcerative colitis [39], celiac disease [40], multiple sclerosis [41], and vitiligo [42]. It should be noted that the lower expression of these MSC and iPSC-enriched genes in whole blood does not rule out important functions in low abundance cell types where expression may be significantly higher. Nevertheless, the contribution of these gene products to the formation of cartilage and bone, and to bone mineralization, together with their enriched expression in MSCs, suggests that disease-specific iPSCs that can be used to generate cell types involved in bone formation (i.e., MSCs, osteoblasts, and chondrocytes) will facilitate important functional genomics studies in AS pathogenesis.

It is noteworthy that Xie et al. recently reported that MSCs obtained from AS patients have an increased osteogenic differentiation capacity, apparently due to an imbalance between BMP2 and Noggin secretion [43]. The underlying cause of these differences is not clear. While genetic differences could play a role, the MSCs were obtained from bone marrow aspirates from patients with active disease, and thus the behavior of the cells might also reflect the influence of disease-related cytokines, other soluble factors, and/or epigenetic differences. The development and application of iPSC technology provides an opportunity to address this question, since iPSCs are reprogrammed, erasing disease-specific epigenetic differences, and thereafter generated under controlled conditions.

In conclusion, we have generated iPSCs from axSpA patients and differentiated them into cell types that are relevant to the pathogenesis of axSpA/AS. We show for the first time that several AS risk genes that are likely to have an impact on osteogenesis are preferentially expressed in iPSC-derived MSCs that serve as precursors to osteoblasts. The ability to maintain and study iPSCs and their derivatives indefinitely provides a unique opportunity to evaluate their function under defined experimental conditions including inflammation or mechanical stress, and to determine their influence on other cell lineages to better understand dysregulated bone formation in axSpA/AS.

## Electronic supplementary material

Supplementary Table 1 (DOCX 13 kb)

ESM 1 (DOCX 93 kb)

## Abbreviations

axSpA: Axial spondyloarthritis
AS: Ankylosing spondylitis
HC: Healthy control
iPSC: Induced pluripotent stem cell
MSC: Mesenchymal stem cell
P1: Patient 1
P2: Patient 2
FBS: Fetal bovine serum
L1: Laboratory 1
L2: Laboratory 2
EB: Embryoid body
DMEM: Dulbecco’s modified Eagle’s medium
MEM-α: Minimal essential medium eagle alpha
E8 medium: Essential E8 medium
E6 medium: Essential E6 medium
TRAP: Tartrate-resistant acidic phosphatase
α-SMA: Smooth muscle actin
AFP: Human alpha-1-fetoprotein
βIII-TUB: βIII tubulin
BSA: Bovine serum albumin
RPKM: Reads per kilobase million
PCA: Principal components analysis
