# Supplementary material for: Generation and differentiation of induced pluripotent stem cells reveal ankylosing spondylitis risk gene expression in bone progenitors
Source: Clin Rheumatol. 2016 Nov 18;36(1):143–54. doi: 10.1007/s10067-016-3469-5 (PMC5216109; doi:10.1007/s10067-016-3469-5)
Supplement: Supplementary file 1 — (DOCX 13 kb) [file 10067_2016_3469_MOESM1_ESM.docx]

**TABLES**

**Supplementary Table 1.** Main pathways differentially expressed in one cell type (iPSCs, MSCs or blood cells) compared to the other two cell types using Ingenuity Pathway Analysis (IPA)

**Gene Expression Pathways Distinguishing iPSCs, MSCs and Peripheral Blood Cells**

**iPSCs**

Role of OCT4 in mammalian stem cell pluripotency

Cell cycle control and replication

Transcriptional regulatory network of embryonic stem cells

**MSCs**

Hepatic fibrosis (actin, collagen expression)

Caveolar mediated endocytosis signaling

Role of osteoblasts, osteoclasts and chondrocytes in RA

Regulation of epithelial-mesenchymal transition pathway

Wnt/β-catenin signaling

**Blood Cells**

Antigen presentation pathway

Natural killer cell signaling

Crosstalk between dendritic cells and natural killer cells

Communication between innate and adaptive immune cells

T helper cell differentiation
