# Supplementary material for: Generation and differentiation of induced pluripotent stem cells reveal ankylosing spondylitis risk gene expression in bone progenitors
Source: Clin Rheumatol. 2016 Nov 18;36(1):143–54. doi: 10.1007/s10067-016-3469-5 (PMC5216109; doi:10.1007/s10067-016-3469-5)
Supplement: Supplementary file 2 — (DOCX 93 kb) [file 10067_2016_3469_MOESM2_ESM.docx]

**Journal: Clinical Rheumatology**

**Generation and Differentiation of Induced Pluripotent Stem Cells Reveal Ankylosing Spondylitis Risk Gene Expression in Bone Progenitors**

Gerlinde Layh-Schmitt^1^*^+^, Shajia Lu^2^*, Fatemeh Navid^1^, Stephen R. Brooks^3^, Emily Lazowick^1^_,_ Kathryn M. Davis^2^, Cristina Montagna^4^, Massimo Gadina^2^, Robert A. Colbert^1^

^1^Pediatric Translational Research Branch, ^2^Translational Immunology Section, ^3^Biodata Mining and Discovery Section, Office of Science and Technology, NIAMS, NIH, Bethesda, MD, USA, ^4^Department of Genetics, Albert Einstein College of Medicine, Bronx, NY, USA.

*denotes equal contribution

^+^ Corresponding author

Gerlinde Layh-Schmitt

Pedriatric Translational Research Branch, NIAMS, NIH

Bldg. 10/CRC, Rm 1-5132

10 Center Drive MSC 1102

Bethesda, MD 20892, USA

Email: [Layhschmittg@mail.nih.gov](mailto:Layhschmittg@mail.nih.gov)

Phone: 301-451-6019

Fax: 301-480-5189

Shajia Lu

Translational Immunology Section, Office of Science and Technology NIAMS, NIH

USA

Email: [lushajia@ep.niams.nih.gov](mailto:lushajia@ep.niams.nih.gov)

Fax: 301-480-6372

Fatemeh Navid

Pediatric Translational Research Branch, NIAMS, NIH

USA

Email: [navidf2@mail.nih.gov](mailto:navidf2@mail.nih.gov)

Fax: 301-480-5189

Stephen R. Brooks

Biodata Mining and Discovery Section, Office of Science and Technology, NIAMS, NIH

USA

Email: [stephen.brooks@nih.gov](mailto:stephen.brooks@nih.gov)

Fax: 301-480-6372

Emily Lazowick

Pediatric Translational Research Branch, NIAMS, NIH

USA

Email: [emily.lazowick@nih.gov](mailto:emily.lazowick@nih.gov)

Fax: 301-480-5189

Kathryn M. Davis

Translational Immunology Section, Office of Science and Technology NIAMS, NIH

USA

Email: [davis.kathrynm@gmail.com](mailto:davis.kathrynm@gmail.com)

Fax: 301-480-6372

Cristina Montagna

Department of Genetics, Albert Einstein College of Medicine

USA.

Email: [cristina.montagna@einstein.yu.edu](mailto:cristina.montagna@einstein.yu.edu)

Fax: 718-678-1016

Massimo Gadina

Translational Immunology Section, Office of Science and Technology NIAMS, NIH

USA

Email: [gadinama@mail.nih.gov](mailto:gadinama@mail.nih.gov)

Fax: 301-480-6372

Robert A. Colbert

Pediatric Translational Research Branch, NIAMS, NIH

USA

Email: [colbertr@mail.nih.gov](mailto:colbertr@mail.nih.gov)

Fax: 301-480-5189

**Supplementary Methods:**

**Generation of induced pluripotent stem cells**

Fibroblasts at passage 2 or 3 were plated on 6-well plates at a density of 1-2 x 10^5^ cells/well. After 24-48 hours, the cells were transduced with Sendai virus (MOI=3) encoding OCT4, SOX2, KLF4 and MYC. On day 7 after transduction, the cells were re-plated on mouse embryonic feeder cells in culture dishes at 0.5-2 x 10^5^ cells per 100 mm dish and cultured in fibroblast medium (DMEM with GlutaMAX-1, 10% FBS, 100 µM MEM Non-Essential Amino Acids (Gibco/Life Technologies). Twenty-four hours later medium was replaced with iPSC medium (KnockOut DMEM/F-12, 20% KnockOut Serum Replacement, 100 µM MEM Non-Essential Amino Acids, 1X GlutaMAX-I supplement, 100 µM β-mercaptoethanol, 4 ng/ml basic FGF) (Gibco/Life Technologies/Life Technologies). Colonies were picked between 12 and 21 days after transduction. Single colony subcloning was performed for at least 10 passages to obtain virus-free clones.

**Expression analysis of Sendai virus transgenes and endogenous pluripotency genes**

Real-time (RT) PCR (RT-PCR) was used to assess expression of Sendai virus transgenes and endogenous pluripotency genes in iPSCs. To examine the loss of Sendai virus transgenes and the expression of endogenous stem cell specific genes in iPSCs, total RNA was purified with Trizol (Invitrogen) and treated with Turbo DNA-free kit (Ambion) to remove potentially contaminating genomic DNA. First-strand cDNA was synthesized from 1 µg of total RNA using random hexamer primers. PCR was performed using the Advantage 2 PCR kit (Clontech). The following amplification cycles were utilized: 1 cycle at 95°C for 1 minute; 30 cycles at 95°C for 30 seconds then 68°C for 1 minute. The primer sets used to examine Sendai virus transgene expression (OCT4, SOX2, KLF4 and MYC) were included in the CytoTune-iPS Reprogramming kit protocol (Invitrogen). For each set, one primer binds to viral sequences while the second primer is specific for the transgene. Primer sets used to detect expression of the endogenous human pluripotency genes are listed are listed below. One primer of each set binds to either the 3’ or 5’ untranslated region of the endogenous gene (not present in the viral transgenes) and the second primer recognizes a coding sequence in the gene.

| Target | primer sets | product size |
| --- | --- | --- |
| OCT3/4 | F' AGTTTGTGCCAGGGTTTTTG | 113 bp |
|  | R' ACTTCACCTTCCCTCCAACC |  |
| NANOG | F' TTTGGAAGCTGCTGGGGAAG | 194 bp |
|  | R' GATGGGAGGAGGGGAGAGGA |  |
| SOX2 | F' GGGAAATGGGAGGGGTGCAAAAGAGG | 151 bp |
|  | R' TTGCGTGAGTGTGGATGGGATTGGTG |  |
| KLF4 | F 'ACGATCGTGGCCCCGGAAAAGGACC | 397 bp |
|  | R' TGATTGTAGTGCTTTCTGGCTGGGCTCC |  |
| MYC | F' GCGTCCTGGGAAGGGAGATCCGGAGC | 330 bp |
|  | R' TTGAGGGGCATCGTCGCGGGAGGCTG |  |

**Embryoid body formation**

Pluripotency of iPSCs was documented by embryoid body (EB) formation and the presence of all three germ layers [[11](#_ENREF_9)]. Induced pluripotent stem cells were harvested using 1 U/ml Dispase (StemCell Technologies), and transferred to ultralow attachment plates in EB medium (DMEM with 20% FBS). After 10 days non-adherent spheroid cell aggregates were plated onto gelatin coated tissue culture dishes and incubated for an additional 9 to 14 days. EBs were fixed in 4% paraformaldehyde (Sigma-Aldrich) and then subjected to immunostaining.

**Karyotyping**

To perform Spectral Karyotyping (SKY), cells were grown to 70% confluence and arrested in mitosis with colcemid (0.1 µg/ml) for 16 hr. Cells were processed for metaphase preparation with standard hypotonic treatment (0.075 M KCl) and fixed in a methanol/acetic acid mixture (3:1). Metaphase chromosomes were dropped in a humidification chamber (Thermotron, Holland, MI) and SKY hybridization was carried out using the Applied Spectral Imaging (Applied Spectral Imaging (ASI), Carlsbad, CA) hybridization probes using the manufacturers recommendations. Images for a minimum of 10 metaphase spreads were acquired with an Olympus BX61 microscope (Olympus, Shinjuku Tokyo Japan) equipped with a spectracube and analyzed using the HiSKY software (ASI). Data were summarized in a table using the international system for human cytogenetic nomenclature (ISCN 2009 <http://www.karger.com/Book/Home/244102>). The image (Figure S2) shown was derived from iPSC (P1L1) chromosomes at passage 31. Similar results (not shown) were obtained at passage 40.

**Differentiation of iPSCs into monocytes/macrophages**

Differentiation was performed in 4 stages. In Stage 1 iPSCs were incubated in APEL media (Stemcell Technologies) containing 30 ng/ml VEGF, 30 ng/ml BMP4, 40 ng/ml SCF and 50 ng/ml Activin A (Stemcell Technologies) for 4 days. In Stage 2, cells were differentiated for 9 days in APEL media containing 300 ng/ml SCF, 300 ng/ml Flt3L, 10 ng/ml IL-3, 10 ng/ml IL-6, 50 ng/ml GCSF and 25 ng/ml BMP4 (Stemcell Technologies) into hematopoietic progenitors. To further expand progenitors, cells were incubated (Stage 3) for 7 days in IMDM supplemented with 10% FBS, 100 U/mL penicillin, 100 μg/mL streptomycin, 5% protein-free hybridoma media (Life Technologies), 0.1 mM β-mercaptoethanol (Gibco/Life Technologies), 100 ng/ml SCF, 100 ng/ml Flt3L, 100 ng/ml IL-6, 10 ng/ml TPO and 10 ng/ml IL-3. In the final stage (Stage 4), cells were cultured in IMDM containing 10% FBS, 100 U/mL penicillin, 100 μg/mL streptomycin and 100 ng/ml M-CSF (Peprotech) for approximately 14 days, with media replaced every 3-4 days. Aliquots of Stage 4 cells were used for FACS analysis to evaluate myeloid specific markers and function.

**Differentiation of iPSC-derived MSCs into chondrocytes, adipocytes, and osteoblasts**

For chondrocyte differentiation MSCs were grown in a 75 cm^2^ flask (Costar) to 70% confluence, detached by TrypLE (Gibco/Life Technologies), and pelleted at 400 x g for 5 min. The pellet was cultured for 3 weeks with chondrocyte differentiation medium (Human Mesenchymal Stem Cell Functional Identification Kit, R&D Systems), with replacement every 3 days. As a control, one pellet was cultured in MEM-alpha. After two weeks cells were stained for chondrocyte specific aggrecan production with alcian blue (Sigma-Aldrich).

For adipocyte differentiation, MSCs were seeded at 10^5^ cells per well in a six well plate. At 100% confluence cells were cultured in adipocyte differentiation medium (Chemicon) for 21 days with medium changes every three days. The cells were fixed with 4% paraformaldehyde and stained with oil red O (Chemicon) to visualize fat droplets.

For osteoblast differentiation, MSCs were seeded at 10^5^ cells/well in 6 well plates or at 0.5 x 10^5^ in 12 well plates and differentiated in osteogenic medium (OS+): MEM-alpha (Gibco/Life Technologies), 15% Hyclone FBS, 100 U/mL penicillin, 100 μg/mL streptomycin, 10mM β-glycerophosphate, 50 µg/mL ascorbic acid, 10 nM dexamethasone (all from Sigma-Aldrich) for 28 days, with medium changed every 2-3 days. For maintaining MSCs and comparative experiments MSCs were cultured in OS- medium (MEM-alpha (Gibco/Life Technologies), 15% Hyclone FBS, 100 U/mL penicillin, 100 μg/mL streptomycin without osteogenic additives. For gene expression experiments, cells were lysed in Trizol at days 0, 4, 7, 11, 17 and 21 after feeding with OS+ medium. For quantification of mineralization, cells were fixed with 4% paraformaldehyde in PBS and then stained with alizarin red (Sigma-Aldrich) (20 minutes in 2% w/v in distilled water, pH 4.1). Plates were air-dried before imaging. For quantitative analysis alizarin red was extracted with 1 ml 10% w/v cetylpyridinium chloride solution in 10 mM sodium phosphate (all from Sigma-Aldrich) at pH 7.0 for 20 min at room temperature. The extracted stain (200 µl) were transferred to 96 well plates and measured in a spectrophotometer at 560 nm.
